# Difficult diagnosis of an air‐containing, abscess‐like, and mass‐like pancreatic head lesion

**DOI:** 10.1002/ccr3.4817

**Published:** 2021-09-26

**Authors:** Antigoni Xenou, Eugenia Vranou, Konstantinos A. Boulas, Maria Nathanailidou, Eytyxia Kyriakidou, Konstantinos Sitaridis, Isaac Filippidis, Anestis Hatzigeorgiadis

**Affiliations:** ^1^ Department of Radiology General Hospital of Drama Drama Greece; ^2^ Department of General Surgery General Hospital of Drama Drama Greece

**Keywords:** complications, diverticulosis, diverticulum, duodenum, pancreatitis

## Abstract

Duodenal diverticulosis can be a difficult CT diagnosis and should be considered in the differential diagnosis when a periduodenal mass‐like structure that may contain air, air‐fluid level, or oral contrast material is depicted.

## Graphical abstract

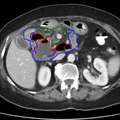

An otherwise healthy 71‐year‐old woman presented due to a 4‐h episode of mild right‐upper quadrant pain. Vital signs were normal and physical examination revealed right‐upper quadrant tenderness. Complete blood count was normal, serum amylase five times elevated, and liver‐associated enzymes mildly elevated. Ultrasonography revealed the presence of biliary microlithiasis, increased pancreatic volume with a marked decrease in echogenicity, and the absence of gallbladder wall thickening, pericholecystic fluid, and biliary tree dilatation. Interestingly, CT revealed a couple of ill‐defined, air‐containing, mass‐like, abscess‐like lesions interposed between duodenum and pancreas along with pancreatic head enlargement and peripancreatic inflammatory changes (Figure 1).

**FIGURE 1:**
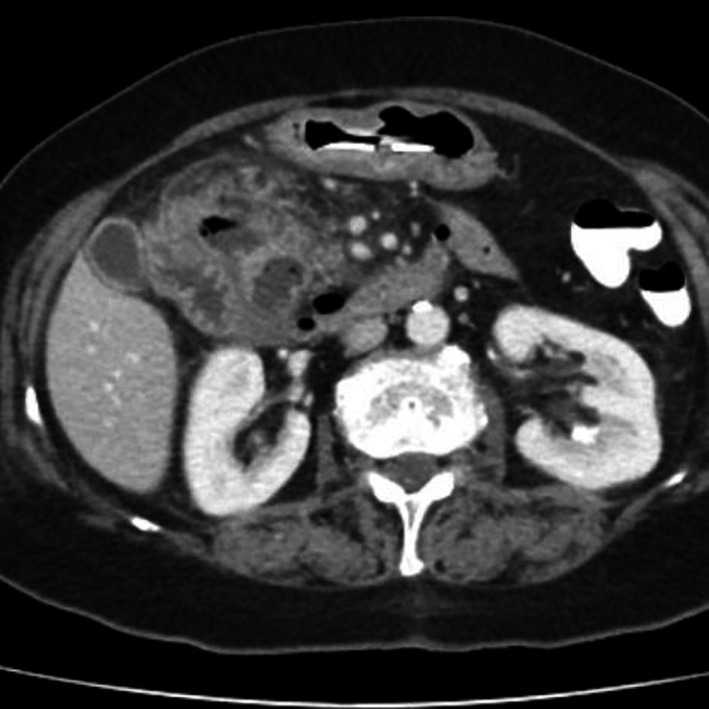
CT revealed a couple of ill‐defined and abscess‐like lesions interposed between duodenum and pancreas along with pancreatic head enlargement and peripancreatic inflammatory changes

## 1 QUIZ QUESTION: WHAT IS YOUR DIAGNOSIS?

**FIGURE 2:**
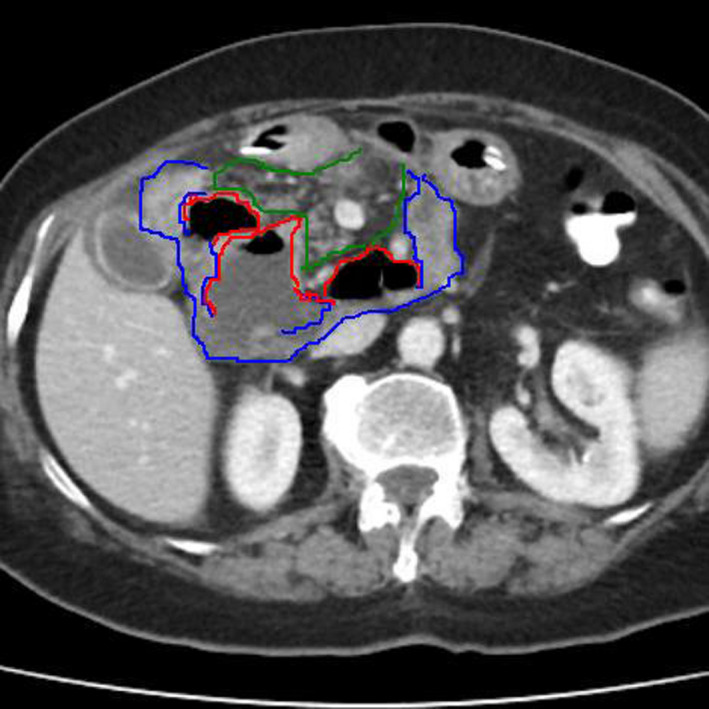
CT review revealed three duodenal diverticula depicted as three ill‐defined, air‐containing, mass‐like, and abscess‐like lesions (red outline) between the duodenum (blue outline) and pancreas (green outline)

**FIGURE 3:**
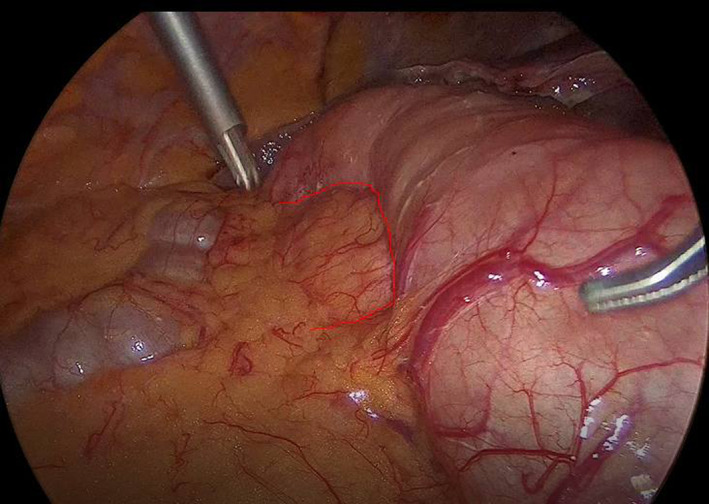
Intraoperative image showing the large third portion duodenal diverticula (red outline)

The presence of periduodenal gas limited differential diagnosis in perforated/penetrating duodenal ulcer, simple/complicated duodenal diverticulosis, pancreatic abscess/infected pseudocyst, and iatrogenic/traumatic duodenal injury. Based on initial assessment, most prominent diagnosis was simple duodenal diverticulosis in the setting of mild acute biliary pancreatitis. Upper endoscopy confirmed the presence of three large duodenal diverticula at medial aspect of second and third portion all with wide neck and normal mucosal appearance, as eventually featured after CT review (Figure 2). The patient managed with early conservative treatment of acute biliary pancreatitis followed by delayed laparoscopic cholecystectomy (Figure 3).

Duodenal diverticula are common and usually asymptomatic. Duodenal diverticulitis is rare due to their large neck size and improved intraluminal flow.^1^ CT appearance includes a saccular outpouching between duodenum and pancreas containing air‐fluid level, contrast material, or debris. Making the correct diagnosis can be challenging, as radiographic features mimic other intra‐abdominal processes, as referred above, and rarely periampullary neoplasms.^2^

## CONFLICT OF INTEREST

The authors declare that they have no conflict of interests.

## AUTHOR CONTRIBUTIONS

All authors equally accessed the data and contributed to the preparation of the manuscript. BKA and HA were equally responsible for making and performing treatment decisions. HA reviewed the manuscript for critical intellectual content and had the final approval.

## STATEMENT OF HUMAN AND ANIMAL RIGHTS

The present article does not contain any studies with human or animal subjects performed by any of the authors.

## CONSENT

Informed consent was obtained from the patient.

## Data Availability

Data sharing is not applicable to this article as no datasets were generated or analyzed during the current study.
